# Fully Automated Quantitative Measurement of Serum Organic Acids via LC-MS/MS for the Diagnosis of Organic Acidemias: Establishment of an Automation System and a Proof-of-Concept Validation

**DOI:** 10.3390/diagnostics11122195

**Published:** 2021-11-25

**Authors:** Yasushi Ueyanagi, Daiki Setoyama, Daisuke Kawakami, Yuichi Mushimoto, Shinya Matsumoto, Taeko Hotta, Dongchon Kang

**Affiliations:** 1 Department of Clinical Chemistry and Laboratory Medicine, Kyushu University Hospital, Fukuoka 812-8582, Japan; ueyanagi.yasushi.964@m.kyushu-u.ac.jp (Y.U.); shmatumo@cclm.med.kyushu-u.ac.jp (S.M.); thotta@cclm.med.kyushu-u.ac.jp (T.H.); 2 Department of Clinical Chemistry and Laboratory Medicine, Graduate School of Medical Sciences, Kyushu University, Fukuoka 812-8582, Japan; 3 Shimadzu Corporation, Kyoto 604-8511, Japan; daikawa@shimadzu.co.jp; 4 Department of Pediatrics, Graduate School of Medical Sciences, Kyushu University, Fukuoka 812-8582, Japan; mushiu1@gmail.com

**Keywords:** liquid chromatography-mass spectrometry, automated sample preparation, organic acid analysis, 3-Nitrophenylhydrazine, organic acid disorders

## Abstract

Gas chromatography-mass spectrometry has been widely used to analyze hundreds of organic acids in urine to provide a diagnostic basis for organic acidemia. However, it is difficult to operate in clinical laboratories on a daily basis due to sample pretreatment processing. Therefore, we aimed to develop a fully automated system for quantifying serum organic acids using liquid chromatography-tandem mass spectrometry (LC-MS/MS). The pretreatment CLAM-2030 device was connected to an LC-MS/MS system for processing serum under optimized conditions, which included derivatizing serum organic acids using 3-Nitrophenylhydrazine. The derivatized organic acids were separated on a reverse-phase Sceptor HD-C column and detected using negative-ion electrospray ionization multiple reaction monitoring MS. The automated pretreatment-LC-MS/MS system processed serum in less than 1 h and analyzed 19 serum organic acids, which are used to detect organic acidemias. The system exhibited high quantitative sensitivity ranging from approximately 2 to 100 µM with a measurement reproducibility of 10.4% CV. Moreover, a proof-of-concept validation of the system was performed using sera from patients with propionic acidemia (*n* = 5), methylmalonic acidemia (*n* = 2), and 3-methylcrotonylglycinuria (*n* = 1). The levels of marker organic acids specific to each disease were significantly elevated in the sera of the patients compared to those in control samples. The automated pretreatment-LC-MS/MS system can be used as a rapid in-hospital system to measure organic acid levels in serum for the diagnosis of organic acidemias.

## 1. Introduction

Newborn screening tests are performed for the early detection of inborn errors in metabolism. Amino acids and acylcarnitines are analyzed in dried blood spot samples, collected from newborns at approximately five days after birth, to determine whether the genes encoding metabolic enzymes are defective [1,2,3]. Newborn screening tests are capable of detecting amino acid and fatty acid disorders; however, they are insufficient to distinguish organic acid disorders. Therefore, additional analyses are required to detect urinary organic acids. Gas chromatography-mass spectrometry (GC-MS) has been widely used to measure more than a hundred kinds of organic acids in urine samples, providing a diagnostic basis for organic acid disorders [4,5,6]. However, GC-MS requires complex sample pretreatment, such as the extraction of organic acids from biological samples, drying of extracted solvents, and reaction with derivatizing reagents, which makes it difficult to operate on a daily basis in a clinical laboratory. It takes more than a week to obtain the test results in Japan, since newborn screenings and organic acid analyses are performed in a limited number of facilities. Organic acid analysis is urgently needed because organic acid disorders cause acute symptoms and prove to be fatal [7,8,9]. Therefore, clinical laboratories require methods to analyze organic acids in a simple, quick, and automated manner while requiring as little human effort as possible.

Liquid chromatography-tandem mass spectrometry (LC-MS/MS) has already been used in many clinical laboratories for a broad range of clinical tests, such as therapeutic drug monitoring (TDM) [10,11] and the analysis of hormones [12,13] and vitamins [14,15], as well as organic acid analysis. However, there is an increasing demand for the employment of LC-MS/MS systems in clinical laboratories for diagnostic purposes. Therefore, the development of an automated system for LC-MS/MS analysis and the pretreatment of clinical samples is warranted.

In this study, we optimized a device that comprises a fully automated pretreatment system, CLAM-2030, directly coupled to LC-MS/MS, which measures serum organic acids and can be used to distinguish organic acid disorders. Using our automated system, it takes less than an hour from setting the blood collection tube to obtaining the result, which is a significant reduction in analysis time from that of conventional GC-MS. Moreover, to validate the clinical utility of the system, a total of 19 organic acids were quantified using our system using the sera of patients with organic acid disorders, including propionic acidemia (PA), methylmalonic acidemia (MMA), and 3-methylcrotonylglycinuria (MCG). Our results suggest that the automated pretreatment-LC-MS/MS system can be practically used as a rapid in-hospital system to measure organic acid levels in the serum.

## 2. Materials and Methods

### 2.1. Chemicals and Reagents

The ultrapure water, formic acid, methanol, and acetonitrile were purchased from Fujifilm Wako Pure Chemicals (Osaka, Japan). The 3-Nitrophenylhydrazine (3-NPH) and *N*-(3-dimethylaminopropyl)-*N*′-ethylcarbodiimide (EDC) hydrochloride were purchased from Sigma-Aldrich (St. Louis, MO, USA). The standards for the target compounds and internal standards used for quantitative analysis are listed in Supplemental Table S1. The ^13^C_3_-3-Hydroxypropionic acid and ^13^C_4_-methylmalonic acid were used as internal standards for 3-hydroxypropionic acid and methylmalonic acid, respectively. For the other compounds, 2-ethylbutyric acid was used as the internal standard.

### 2.2. Optimizing Multiple Reaction Mode (MRM) Transitions of 3-NPH-Derivatized Organic Acids

Standard organic acids were dissolved in 90% methanol to obtain a concentration of 10 µM, 100 µL of which were added with 25 µL of 50 mM 3-NPH (50% methanol solution) and 25 µL of 50 mM EDC/9% pyridine (50% methanol solution) and incubated for 15 min at 23 °C. Two microliters of each solution were infused directly into the MS system to obtain optimum conditions for MRM transition in the negative mode (Table 1).

### 2.3. Sample Preparation

All the sample deproteinization and derivatization reactions were performed using the fully automated LCMS pretreatment system CLAM^TM^-2030 (Shimadzu Corp., Kyoto, Japan). Mass spectrometry was performed using a high-performance liquid chromatography-mass spectrometry LCMS^TM^-8040 system (Shimadzu Corp., Kyoto, Japan) coupled with CLAM-2030.

In CLAM-2030, a dedicated filter consisting of a polytetrafluoroethylene membrane with a pore size of 0.45 µm and vials were used for pretreatment. First, a total of 20 µL of methanol was dispensed onto the filter to activate the dedicated filter with hydrophobic properties. Next, 10 µL of serum sample and 90 µL of methanol containing the internal standard were dispensed into a dedicated vial and stirred for 60 s. The samples were filtered using vacuum pressure (approximately 50 to 60 kPa) for 60 s in the filtration unit and collected in a collection vial. The collected deproteinized samples were derivatized by adding 25 µL of 200 mM 3-NPH (50% methanol solution) and 25 µL of 200 mM EDC/9% pyridine (50% methanol solution), stirred for 30 s, followed by incubation at room temperature (RT; 23 °C) for 15 min. The derivatized sample was then transferred to the autosampler of the LC-MS/MS system, and 10 µL were injected.

This method required 30.2 min for pretreatment using CLAM-2030 and 24 min for LC-MS/MS analysis. Since CLAM-2030 performs sequential processing, it is possible to start the next pretreatment during LC-MS/MS analysis. The sample was injected at intervals of 29 min, including the interval between CLAM-2030 and LC-MS/MS.

### 2.4. LC-MS/MS

A Shim-pack Scepter HD-C18-80 column (150 × 2.1 mm, 3 μm; Shimadzu) was used for the sample separation. The mobile phase comprised (A) 0.1% aqueous formic acid and (B) acetonitrile at a flow rate of 0.35 mL/min. The gradient used was as follows: 0.00–6.00 min, 16–25% (B); 6.00–18.00 min, 25–70% (B); 18.01–21.00 min, 95% (B); 21.01–24.00 min, 16% (B). The oven temperature was set to 40 °C.

The mass spectrometer was operated in the negative electrospray ionization mode and MRM. The interface settings were as follows. Nebulizing-gas flow rate: 3.0 L/min; drying-gas flow rate: 15.0 L/min; desolvation line temperature: 250 °C; heat block temperature: 400 °C; collision gas pressure: 230 kPa. The dwell time was set to 22.0–163.0 ms to obtain at least 20 data points across the peak. All the peak analyses for the LC-MS/MS were performed using LabSolutions ver. 5.97 SP1 (Shimadzu), and the peak area was used as the signal intensity.

**Figure 1:**
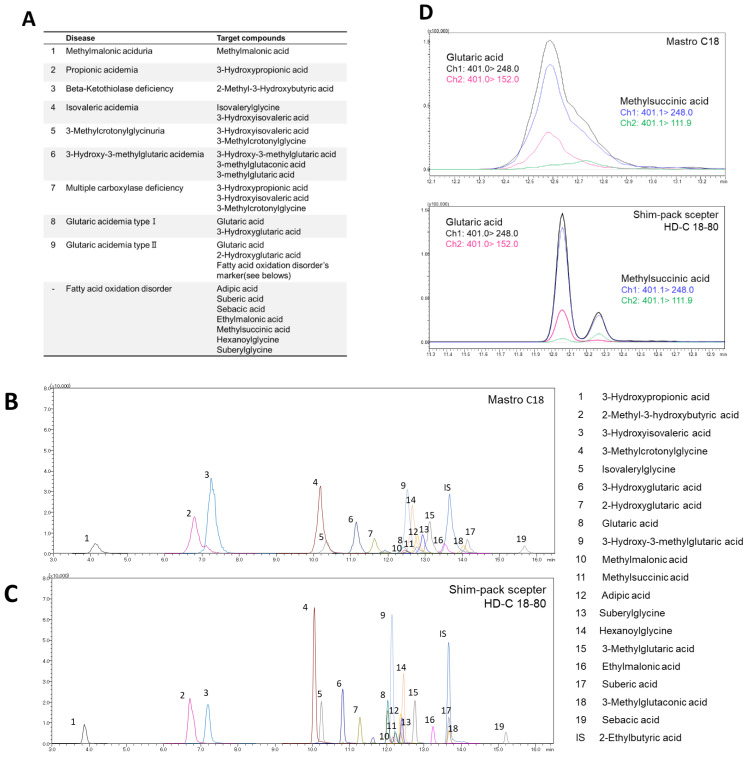
Investigation of LC separation conditions. (**A**) Targeted diseases and marker metabolites. MRM chromatogram of 3-NPH derivatized organic acids obtained from (**B**) MastroC18 and (**C**) Shim-pack sceptor HD-C18-80. (**D**) MRM chromatogram of glutaric acid and methylsuccinic acid on MastroC18 and Shim-pack sceptor HD-C18-80 column.

Figure 1A lists the organic acid metabolism disorders that are diagnosed based on the compounds quantified in this analysis, and Table 1 shows the MRM conditions for each compound.

### 2.5. Organic Acid Extraction Conditions for Serum Samples

The samples were spiked with 25 µM of the analyte before or after the extraction of organic acids from the serum samples and then analyzed; the peak area ratio of the analyte to the internal standard was determined. The recovery was calculated as the ratio of the peak area of the sample spiked before extraction to that of the sample spiked after extraction. Extraction using acetonitrile and methanol was performed thrice using each solvent. The data were analyzed by Student’s *t*-test. The *p* values < 0.05 were considered statistically significant.

### 2.6. Optimization of 3-NPH Derivatization Condition in Serum

To optimize the 3-NPH derivatization conditions, a serum sample spiked with 25 µM of the analyte was used for validation. The concentration of 3-NPH was validated at 50, 100, and 200 mM concentrations. The reaction temperatures were tested at RT (23 °C), 35 °C, 45 °C, and 55 °C. The reaction times were tested at 5, 10, 15, and 30 min. The peak areas obtained from the analysis were used to compare the reaction efficiency for each condition.

### 2.7. Method Validation

Linearity: The calibration curve was generated via linear regression of the relative peak area ratios against the five concentrations (2, 10, 20, 50, and 100 µM) with a weighting of 1/x^2^.

Sensitivity: The low limit of detection (LLOD) was defined as the minimum concentration at which the signal/noise ratio was greater than 3.0 in the analysis of the dilution series from 0.063 to 2 µM. The low limit of quantification (LLOQ) was defined as the lowest concentration point at which the error between the concentration obtained via back-calculation from the calibration curve and the actual concentration was less than 20%.

Accuracy and imprecision: simultaneous reproducibility (*n* = 5) and inter-day reproducibility (*n* = 5) were evaluated using two concentrations (5 and 25 µM) of the standard mixture. For accuracy, the mean value of the quantitative concentrations obtained via the analysis of simultaneous reproducibility was calculated, and an error of 15% or less than that of the target value was considered acceptable. For reproducibility, the percentage coefficient of variation (CV%) values were calculated from the quantitative concentrations obtained in each of the intra-assays and inter-assays, and were considered acceptable if they were within 15%.

### 2.8. Proof-of-Concept Validation

Patients and control subjects were recruited by either the Department of Pediatrics or the Department of Clinical Chemistry and Laboratory Medicine of Kyushu University Hospital, respectively. Serum samples from 8 patients (5 patients with PPA, 2 with MMA, and 1 with MCG), who were diagnosed using the conventional GC-MS method using urine samples, and serum samples from a control group of 20 adult males and females were analyzed. For each disease, the concentration values of the marker metabolites were compared to those of the control group.

All the samples were obtained from patients who gave informed consent, and the samples were analyzed in accordance with the protocols approved by the Ethics Committee of Kyushu University (license number: 2020-159).

## 3. Results

### 3.1. Optimization for MS Detection Parameters for 3-NPH Derivatized Organic Acids

In this study, we developed an automated LC-MS/MS-based method to differentiate nine types of organic acidemia (Figure 1A). Derivatization with 3-Nitrophenylhydrazine (3-NPH), which reacts with the carboxylic acids of organic acids in aqueous condition, was chosen to improve the separation and detection sensitivity in our LC-MS/MS system. We optimized the MS detection parameters and MRM transitions (Table 1), in which the precursor ion of the derivatized product was determined based on the compounds, in which all the carboxyl groups were 3-NPH-derivatized (Table 1).

### 3.2. Optimization of LC Separation Conditions

Next, to optimize the LC separation conditions of the 3-NPH-derivatized organic acids, two reverse-phase columns, the conventional MastroC18 and the high-density type Shim-pack Sceptor HD-C18-80 (Sceptor), were evaluated, expecting the latter to show better separation efficacy. As expected, compared to the MRM chromatogram obtained using MastroC18, the width of each peak obtained using the Sceptor column was narrower and sharper (Figure 1B,C). In addition, the Sceptor column was able to separate the structural isomers glutaric acid and methylsuccinic acid (Figure 1D) that could not be separated using the MastroC18 column. Moreover, 3-hydroxypropionic acid and lactic acid (Supplemental Figure S1A) and methylmalonic acid and succinic acid (Supplemental Figure S1B) could be separated, improving the specificity of their compounds to be measured. Therefore, the Sceptor column was used for the separation of 3-NPH-derivatized organic acids.

### 3.3. Extraction Conditions for Serum Organic Acids

**Figure 2:**
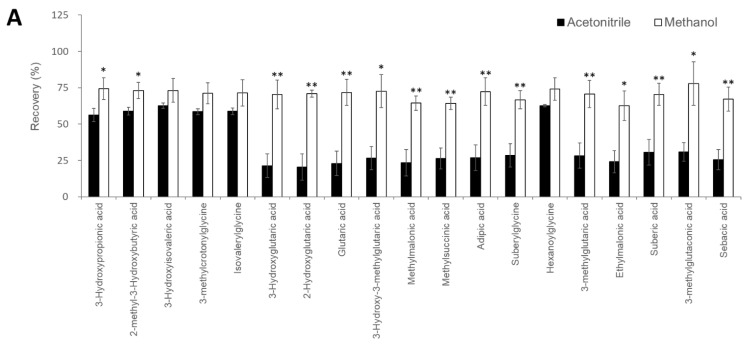

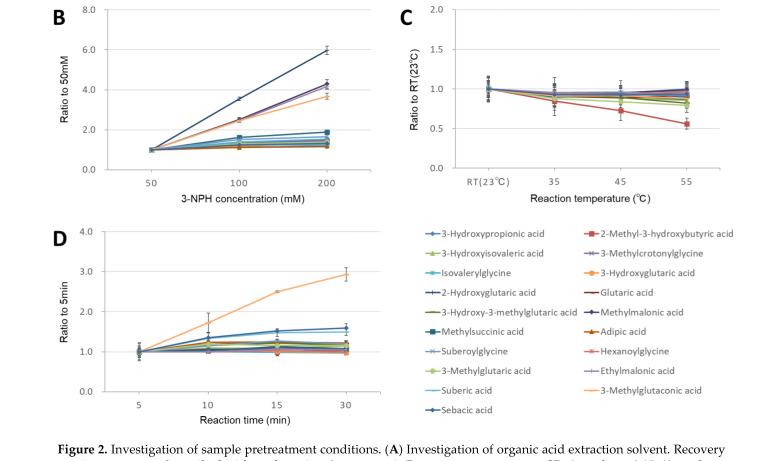
Investigation of sample pretreatment conditions. (**A**) Investigation of organic acid extraction solvent. Recovery rate in serum samples spiked with analytes (*n* = 3 per group). Data represents means ± SD. * *p* value < 0.05, ** *p* value < 0.01 versus Acetonitrile. Effect of 3-NPH derivatization reaction by (**B**) 3-NPH concentration, (**C**) reaction temperature, and (**D**) reaction time. Data represents means ± SD.

The automatic pretreatment device, CLAM-2030 requires the optimization of the deproteinization process and the subsequent 3-NPH derivatization of serum organic acids. First, we compared the extraction efficiency of two solvents, methanol and acetonitrile, for deproteination and extraction of the 19 serum organic acids used to distinguish 9 types of organic acid disorders (Figure 1A). The recovery rate of 25 µM of the analytes spiked in serum samples before and after extraction is shown in Figure 2A. In the acetonitrile fraction, many compounds were found to exhibit a recovery rate lower than 40%, whereas in the methanol fractions, all the compounds showed better recovery than that obtained using acetonitrile; the lowest recovery rate obtained using methanol was 63% for ethylmalonic acid. Therefore, methanol was used in the deproteinizing solution for subsequent organic acid analysis.

### 3.4. Optimization of the 3-NPH Derivatization Reaction

**Figure 3:**
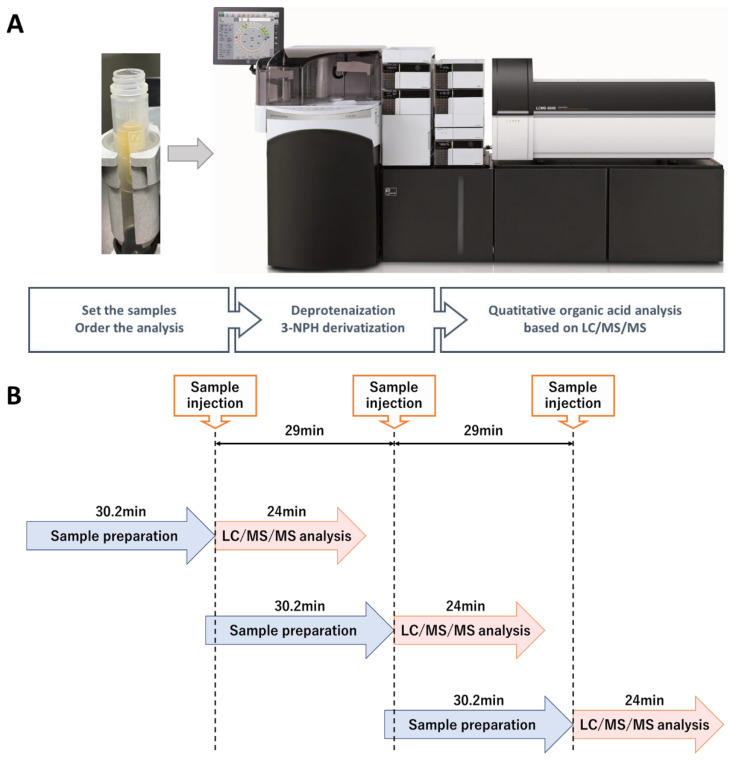
Fully automated organic acid analysis system. (**A**) Schema of the analysis system. (**B**) Flow of sample measurement. The next sample pretreatment can be performed during LCMS analysis.

We optimized the conditions of 3-NPH derivatization, including the concentration of 3-NPH (50 to 200 mM), reaction temperature, and reaction time. The optimal 3-NPH concentration was determined to be 200 mM as it was the maximum concentration that could be stably prepared (Figure 2B). Next, the reaction temperatures of the individual peak areas were examined ranging from RT to 55 °C. For most organic acids, the reaction temperature did not significantly affect the signal intensities, except for that of the 2-methyl-3-hydroxy butyric acid, which showed a clear decrease in the peak area at high temperature. Therefore, the reaction temperature was maintained at 23 °C (Figure 2C). Finally, the reaction time was examined from 5–30 min. Most organic acids showed the same signal intensity for reactions proceeding for 30 min as that of the reaction proceeding for 15 min (Figure 2D). Therefore, the reaction time was set to 15 min to complete the sample pretreatment in a time period close to the sample injection interval of 29 min for LC-MS/MS analysis (Figure 3).

### 3.5. Linearity, Sensitivity, Accuracy, and Imprecision

The quantification parameters of our system for 19 serum organic acids were determined (Table 2). The correlation coefficients of the calibration curves ranging from 2–100 µM were more than 0.992 for all analytes. The LLOD was 0.06 µM for most analytes and was detectable for all analytes from a concentration of at least 0.50 µM (methylsuccinic acid). The accuracy of the assay over five consecutive measurements was adequate (within 15%), with a maximum error of 14.1% at 5 µM and 7.5% at 25 µM. Simultaneous reproducibility (*n* = 5) and inter-day reproducibility (*n* = 5) were 10.4 and 5.7% CV, respectively (both within 15%).

### 3.6. Proof-of-Concept Validation

**Figure 4:**
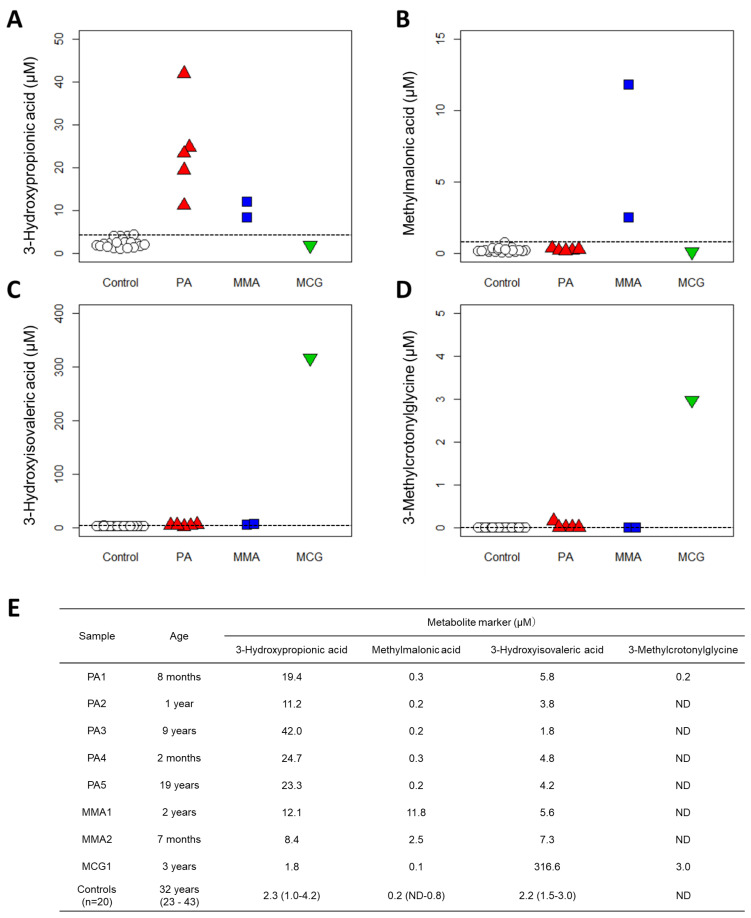
Results of clinical validation. Quantification results with new method from 20 controls and 8 patients: (**A**) 3-Hydroxypropionic acid, (**B**) Methylmalonic acid, (**C**) 3-Hydroxyisovaleric acid and (**D**) 3-Methylcrotonylglycine. PA: propionic acidemia. MMA: methylmalonic acidemia. MCG: methylcrotonylglycinuria. Dashed lines indicate the maximum value of controls. (**E**) Patient data and control data (medians (ranges)). ND: not detected, plotted as 0 µM.

Finally, we evaluated the clinical utility of the automated system using serum samples from nine patients with organic acid disorders including PA (*n* = 5), MMA (*n* = 2), and MCG (*n* = 1). They were follow-up patients who had been diagnosed based on the results of GC-MS analysis of their urine (Table S2). Sera collected from 20 adult male and female participants were used as control samples. For each disease, the concentration values of the marker metabolites in the patient samples were compared to those of the healthy participants (Figure 4).

PA is characterized by an increase in 3-hydroxypropionic acid levels in the blood, but not of methylmalonic acid [16,17]. 3-Hydroxypropionic acid levels in the sera of patients with PA were found to be in the range of 11.2–42.0 µM, which was considerably higher than 1.0–4.2 µM in the control group. By contrast, serum methylmalonic acid levels in patients with PA were almost the same as those in the control group. MMA is characterized by an increase in methylmalonic acid levels in the blood [16,17]. The methylmalonic acid levels were measured as 11.8 µM and 2.5 µM in the sera of two patients with MMA, which were considerably higher than 0.8 µM in the control group. MCG is characterized by an increase in 3-hydroxyisovaleric acid and 3-methylcrotonylglycine levels in the blood [18]. The serum 3-hydroxyisovaleric acid level was measured as 316.6 µM in 1 patient with MCG, compared to 1.5–3.0 µM in the control group, and the 3-methylcrotonylglycine level was 3.0 µM compared to ND in the control group. These data support the diagnosis of the patients, suggesting that quantitative measurement of serum organic acids via automated LC-MS/MS can be used as a diagnostic basis for PA, MMA, and MCG.

## 4. Discussion

We developed a fully automated LC-MS/MS-based analytical system to quantify serum organic acids, providing a diagnostic basis for metabolic disorders such as organic acidemias. The optimization of the pretreatment process using CLAM-2030 (Figure 2), LC separation (Figure 1), and MS detection (Table 1) improved the performance of the system, which was sufficient in terms of the measurement’s reproducibility (Table 2). We also demonstrated that the levels of organic acid markers in the serum samples of patients were elevated compared to those in healthy subjects (Figure 4). These results strongly suggest that our system is reliable in clinical settings, although further improvements are required.

Recently, the use of LC-MS has become widespread in clinical laboratories [19]; however, it is necessary to automate and standardize the pretreatment process while requiring as little human labor as possible to operate it on a daily basis [20,21]. CLAM-2030, a pretreatment module that is coupled with LC-MS, has been used in various clinical applications, such as blood TDM and toxin-drug detection [22]. However, the previously described pretreatment process includes only simple deproteinization and does not involve other complex operations. In this study, we optimized the process of the CLAM-2030 module, including not only deproteinization but also the derivatization of the deproteinized sample using 3-NPH (Figure 3A). As thousands of low-molecular-weight compounds are present in blood [23,24], and their chemical properties vary widely, the efficiency of extraction depends on the solvent used [25,26]. Methanol is commonly used as a deproteinization reagent and has excellent extraction efficiency for various metabolites present in blood samples [25,26]. We confirmed that the extraction efficiency of 19 serum organic acids using methanol was superior to when using acetonitrile (Figure 2A). In addition, we performed derivatization using 3-NPH to measure the organic acid levels using LCMS. Generally, organic acids containing carboxyl group(s) are difficult to separate via LC and show a low sensitivity in MS measurements [27]; therefore, direct analysis via LCMS is considered challenging. Therefore, several attempts were made to derivatize organic acids using specific reagents, followed by separation via LC, and the improvement of their sensitivity via MS. Derivatization using 3-NPH has already been reported to be effective in the simultaneous analysis of cellular citric acid cycle intermediates [28] and fecal short-chain fatty acids [29]. In addition, it was recently reported that it can be applied to the analysis of serum acylcarnitines [30], the compounds that are measured in newborn mass screening [1,2,3]. Thus, it is possible to analyze acylcarnitines and organic acids using our automated analytical system.

In addition to 3-NPH, 2-picolylamine and EDC (and others) are used as derivatizing reagents for carboxylic acids [31,32,33]. While the 3-NPH reaction proceeds in an aqueous solution, reactions with other reagents proceed only in organic solvents. Therefore, liquid-liquid extraction (LLE) and solid-phase extraction (SPE) are required along with protein removal to purify the target compounds in the blood, which is laborious. Thus, we believe that 3-NPH derivatization provides an advantage for the CLAM-2030 system.

At present, the GC-MS system is most commonly used for urinary organic acid analysis [4,5,6]. However, pretreatment of GC-MS urine samples is time-consuming and laborious, requiring urease treatment prior to trimethylsilylation (TMS) or tert-butyldimethylsilylation (TBDMS) derivatization [5,6]. These complex processes can affect the reproducibility of the results. Ohie et al. reported the reproducibility of organic acid analysis using GC-MS with CV values in the range of 8.8–16.1% for TMS derivatization and 4.3–11.0% for TBDMS derivatization [6]. By contrast, our study showed that the reproducibility of the automated LC-MS/MS system was approximately 10.4% CV (Table 2), highlighting its effectiveness and high reproducibility.

One of the major advantages of using LC-MS/MS in clinical laboratories is the ability to analyze multiple components simultaneously. Similar to organic acids, steroid compounds (hormones, etc.) in the blood are suitable for simultaneous analysis, but this has not yet been automated. Conventionally, steroid hormones are individually (or manually) measured by radioimmunoassay (RIA) or their TMS derivatives are measured by GC-MS [34]. TMS derivatization targets the hydroxyl group of a steroid, but the derivative compound is usually unstable. Furthermore, blood hormones that are present in limited amounts are difficult to measure due to their low sensitivity. The derivatization of steroids using picolinic acid (pyridine-2-carboxylic acid) dramatically increases the sensitivity for LC-MS/MS measurements [35,36]. However, this process requires complicated steps such as the LLE of blood steroids and the SPE of their derivatives. Currently, however, the CLAM-2030 system cannot process these steps, but if the function can be extended in the future, the fully automated analysis of steroid compounds will be possible.

We validated our system using sera from patients with three organic acidemias and observed that the serum levels of marker organic acids were higher in the patient samples than in the samples of healthy subjects (Figure 4). These results strongly suggest that measuring serum organic acid levels can provide a sufficient basis for diagnosis. Urinary organic acid analysis via GC-MS can measure hundreds of organic acids and produce rather qualitative results for diagnosis [37]. However, LC-MS has the advantage of producing highly quantitative data; therefore, if the concentration range of each organic acid in the serum of healthy subjects (also known as “reference interval”) can be determined, then quantitative evaluation will be possible not only for diagnosing diseases but also for monitoring the therapeutic effect or prognosis. Moreover, compared to analyzing urine samples that are less invasive but often difficult to collect from newborns and infants [38,39], the use of blood or serum samples is advantageous in clinical practice, especially in situations where tests are urgently needed. 

## 5. Conclusions

To our knowledge, this is the first study to provide the analytical methodology of fully automated LC-MS/MS for the diagnosis of organic acid disorders. However, our pilot study has a few limitations. First, the sample number of patients with organic acid acidemias was small, and the control samples used in this study were collected from adults, despite most test samples being collected from newborns or children (Figure 4E). As most of the patients who undergo organic acid analysis are either newborns or infants, it is important to increase the number of age-matched case-control samples, and to determine the thresholds for the serum levels of each organic acid to identify patients with organic acid disorders. Practically, however, we recognize that the target compounds in our measurement system were insufficient to detect all kinds of organic acids that can be measured by GC-MS. Since this study is only a proof-of-concept study, it will be necessary in the future to develop our measurement system to cover organic acid disorders more comprehensively while adding target compounds. It is also important to create an algorithm that supports the diagnosis of metabolic disorders and to implement it in the automated system. This will lead to the construction of a bona fide, fully automated measurement and diagnostic support system (Supplemental Figure S2). We are currently performing a large-scale demonstration study while improving the automated and integrated analysis system, including the detection of organic acids, amino acids, and acylcarnitines.

## Figures and Tables

**Table 1:** Multiple reaction monitoring transitions of 3-Nitrophenylhydrazine (3-NPH) derivatized organic acids. For all compounds, 3-NPH derivatized reaction was found to occur in all carboxyl groups.

Compounds	Number of Carboxyl Groups	Number of 3-NPH Derivatized	Precursor Ion (*m*/*z*)	Product Ion (*m*/*z*)		Retention Time (min)
				Quantitation	Reference	
3-Hydroxypropionic acid	1	1	224.2	194.1	152.0	3.82
2-Methyl-3-hydroxybutyric acid	1	1	252.0	208.1	165.1	6.75
3-Hydroxyisovaleric acid	1	1	252.2	194.0	152.0	7.23
3-Methylcrotonylglycine	1	1	291.0	209.0	137.0	10.11
Isovalerylglycine	1	1	293.0	137.0	209.0	10.30
3-Hydroxyglutaric acid	2	2	417.0	222.0	178.0	10.87
2-Hydroxyglutaric acid	2	2	417.0	137.0	220.0	11.33
Glutaric acid	2	2	401.0	152.0	248.0	12.08
3-Hydroxy-3-methylglutaric acid	2	2	431.0	236.1	178.0	12.19
Methylmalonic acid	2	2	387.0	178.0	150.0	12.22
Methylsuccinic acid	2	2	401.1	248.0	111.9	12.28
Adipic acid	2	2	415.0	178.0	262.1	12.42
Suberylglycine	2	2	500.2	137.0	209.1	12.44
Hexanoylglycine	1	1	307.1	137.0	208.9	12.49
3-Methylglutaric acid	2	2	415.1	262.1	152.0	12.80
Ethylmalonic acid	2	2	401.1	178.0	150.0	13.29
Suberic acid	2	2	443.2	152.0	137.0	13.71
3-Methylglutaconic acid	2	2	413.0	260.0	178.0	13.72
Sebacic acid	2	2	471.1	137.0	152.0	15.23
^13^C_3_-3-Hydroxypropionic acid (ISTD)	1	1	226.8	196.0	152.0	3.82
^13^C_4_-Methylmalonic acid (ISTD)	2	2	391.2	151.0	179.1	12.22
2-Ethylbutyric acid (ISTD)	1	1	250.2	137.0	107.0	13.71

**Table 2:** Result of validation study. r^2^: correlation coefficient of the calibration curves ranging 2–100 µM. LLOD: Low limit of detection. LLOQ: Low limit of quantification.

Compounds	r^2^	LLOD (µM)	Accuracy (%)		Intra-Assay (*n* = 5) (CV%)		Inter-Assay (*n* = 5) (CV%)	
			5 µM	25 µM	5 µM	25 µM	5 µM	25 µM
3-Hydroxypropionic acid	0.998	0.13	113.6	96.8	0.9	1.8	4.5	4.6
2-Methyl-3-hydroxybutyric acid	0.998	0.06	100.8	93.7	4.3	4.6	3.1	4.6
3-Hydroxyisovaleric acid	0.996	0.25	99.9	95.4	5.5	5.3	3.4	3.6
3-Methylcrotonylglycine	0.996	0.06	100	94.9	1.8	2.0	5.6	4.3
Isovalerylglycine	0.997	0.06	101.6	100.6	3.4	3.4	5.2	3.5
3-Hydroxyglutaric acid	0.994	0.06	96.3	94.0	3.7	1.8	5.3	3.6
2-Hydroxyglutaric acid	0.998	0.06	96.5	92.5	4.6	1.7	4.1	5.7
Glutaric acid	0.999	0.13	101.5	97.4	3.9	1.8	8.6	5.5
3-Hydroxy-3-methylglutaric acid	0.998	0.06	87.2	94.7	6.0	3.6	9.3	3.7
Methylmalonic acid	0.999	0.06	110.4	97.2	4.8	5.2	9.0	4.9
Methylsuccinic acid	0.997	0.50	102.8	101.0	6.5	2.3	4.0	1.8
Adipic acid	0.997	0.13	103.9	96.4	5.1	2.0	7.9	4.1
Suberylglycine	0.999	0.06	108.9	103.1	2.1	4.1	10.4	3.0
Hexanoylglycine	0.998	0.06	109.0	100.3	4.2	3.6	5.3	3.2
3-Methylglutaric acid	0.999	0.06	105.0	100.1	2.9	1.9	3.7	2.2
Ethylmalonic acid	0.992	0.13	94.7	99.5	3.0	3.9	10.2	5.6
Suberic acid	0.997	0.06	114.1	100.6	2.3	4.3	6.0	2.3
3-Methylglutaconic acid	0.999	0.06	106.3	97.8	3.3	3.5	7.1	3.5
Sebacic acid	0.999	0.50	109.6	99.5	2.2	3.5	8.8	3.2

## Data Availability

The data presented in this study are described within the article.

## Supplementary Materials

The following are available online at https://www.mdpi.com/article/10.3390/diagnostics11122195/s1. Figure S1: Structural isomers separation; Figure S2: Schema of automated diagnosis system for organic acid disorders; Table S1: Standard and internal standards; Table S2: Results of urine analysis by GC-MS at the time of diagnosis.
