# Supplementary material for: Fully Automated Quantitative Measurement of Serum Organic Acids via LC-MS/MS for the Diagnosis of Organic Acidemias: Establishment of an Automation System and a Proof-of-Concept Validation
Source: Diagnostics (Basel). 2021 Nov 25;11(12):2195. doi: 10.3390/diagnostics11122195 (PMC8700112; doi:10.3390/diagnostics11122195)
Supplement: Supplementary file 1 [file diagnostics-11-02195-s001.zip › Table S1.pdf]

Supplemental Table1

Standards and internal standards

	No	Name	Vendor	Product No.
Standards	1	3-Hydroxypropionic acid	Tokyo Chemical Industry (Tokyo, Japan)	H0297
	2	Methylmalonic acid	Sigma-Aldrich (St. Louis, MO, USA)	M54058
	3	N-Isovalerylglycine	Sigma-Aldrich (St. Louis, MO, USA)	43424
	4	β-Hydroxyisovaleric acid	Tokyo Chemical Industry (Tokyo, Japan)	H0701
	5	3-Methylcrotonylglycine	Santa Cruz Biotechnology (Dallas, TX, USA)	sc-209651
	6	3-Hydroxy-3-methylglutaric acid	Tokyo Chemical Industry (Tokyo, Japan)	H0436
	7	3-Hydroxy-2-methylbutanoic acid	Toronto Research Chemicals (Toronto, Canada)	H946485
	8	Glutaric acid	Tokyo Chemical Industry (Tokyo, Japan)	G0069
	9	3-Methylglutaconic acid	Sigma-Aldrich (St. Louis, MO, USA)	6689
	10	3-Methylglutaric acid	Tokyo Chemical Industry (Tokyo, Japan)	M0330
	11	3-Hydroxyglutaric acid	Sigma-Aldrich (St. Louis, MO, USA)	4725
	12	2-Hydroxyglutaric acid	Sigma-Aldrich (St. Louis, MO, USA)	90790
	13	Ethylmalonic acid	Fujifilm Wako Pure Chemicals (Osaka, Japan)	320-92121
	14	Methylsuccinic acid	Tokyo Chemical Industry (Tokyo, Japan)	M0430
	15	Adipic acid	Tokyo Chemical Industry (Tokyo, Japan)	A0161
	16	N-Hexanoylglycine	Sigma-Aldrich (St. Louis, MO, USA)	93544
	17	Suberic acid	Santa Cruz Biotechnology (Dallas, TX, USA)	sc-208404
	18	Sebacic acid	Fujifilm Wako Pure Chemicals (Osaka, Japan)	196-00272
	19	N-Suberoylglycine	Sigma-Aldrich (St. Louis, MO, USA)	52888
Internal Standards	1	$^{13}C_3$-3-Hydroxypropionic acid	ALSACHIM (Illkirch, France)	C8223
	2	$^{13}C_4$-Methylmalonic acid	ALSACHIM (Illkirch, France)	C8325
	3	2-Ethylbutyric acid	Sigma-Aldrich (St. Louis, MO, USA)	109959
