# Supplementary material for: Fully Automated Quantitative Measurement of Serum Organic Acids via LC-MS/MS for the Diagnosis of Organic Acidemias: Establishment of an Automation System and a Proof-of-Concept Validation
Source: Diagnostics (Basel). 2021 Nov 25;11(12):2195. doi: 10.3390/diagnostics11122195 (PMC8700112; doi:10.3390/diagnostics11122195)
Supplement: Supplementary file 1 [file diagnostics-11-02195-s001.zip › Table S2.pdf]

Supplemental Table2

Results of urine analysis by GC-MS at the time of diagnosis.

Patient	GC-MS (Urine)	
	Age at sampled	Results (normal range)
PA1	1 month	3-Hydroxypropionic acid: 3.5 (0.0–1.1)
		Phosphoric acid: 145.6 (0.0–43.0)
		Hexanoylglycine: 2.3 (0.0)
		Methylcitric acid (1): 6.5 (0.0–1.1)
		Methylcitric acid (2): 5.8 (0.0–1.0)
PA2	6 months	Glycolic acid: 5.9 (0.0–2.2)
		3-Hydroxypropionic acid: 7.6 (0.0–1.1)
		3-Hydroxybutyric acid: 14.0 (0.0–3.7)
		Benzoic acid: 70.9 (0.0–18.7)
		3-Hydroxyglutaric acid: 0.9 (0.0)
		Hexanoylglycine: 2.5 (0.0)
		Vanillic acid: 6.9 (0.0)
		Citric acid: 1180.9 (31.4–572.0)
		Methylcitric acid (1): 1.3 (0.0–1.1)
		Methylcitric acid (2): 2.2 (0.0–1.0)
PA3	NA	NA
PA4	1 month	Oxalic acid: 5.8 (0.0)
		Fumaric acid: 12.1 (0.0–7.3)
		Malic acid: 2.1 (0.0–0.7)
		5-Hydroxymethyl-2-furoic acid: 81.8 (0.0)
		2-Ketoglutaric acid (1): 184.0 (3.0–102.9)
		Hexanoylglycine: 8.6 (0.0)
		Methylcitric acid (1): 2.5 (0.0–1.1)
		Methylcitric acid (2): 2.8 (0.0–1.0)
PA5	18 years	Methylcitric acid
		3-Hydroxypropionic acid
		Propionylglycine
		Tiglylglycine
MMA1	1 month	Methylmalonic acid
		Pyruvic acid
		Lactic acid
		Ethylmalonic acid
MMA2	20 days	3-Hydroxypropionic acid: 1.8 (0.0–1.1)
		Pyruvic acid: 36.0 (0.0–24.1)
		Methylmalonic acid: 135.7 (0.2–3.6)
		Fumaric acid: 61.0 (0.0–7.3)
		Malic acid: 7.0 (0.0–0.7)
		Hexanoylglycine: 2.9 (0.0)
		Vanillic acid: 12.3 (0.0)
		Methylcitric acid (1): 3.2 (0.0–1.1)
		Methylcitric acid (2): 3.0 (0.0–1.0)
MCG1	NA	NA
