# Supplementary material for: Fully Automated Quantitative Measurement of Serum Organic Acids via LC-MS/MS for the Diagnosis of Organic Acidemias: Establishment of an Automation System and a Proof-of-Concept Validation
Source: Diagnostics (Basel). 2021 Nov 25;11(12):2195. doi: 10.3390/diagnostics11122195 (PMC8700112; doi:10.3390/diagnostics11122195)
Supplement: Supplementary file 1 [file diagnostics-11-02195-s001.zip › Figure S1.pdf]

Supplemental Figure1

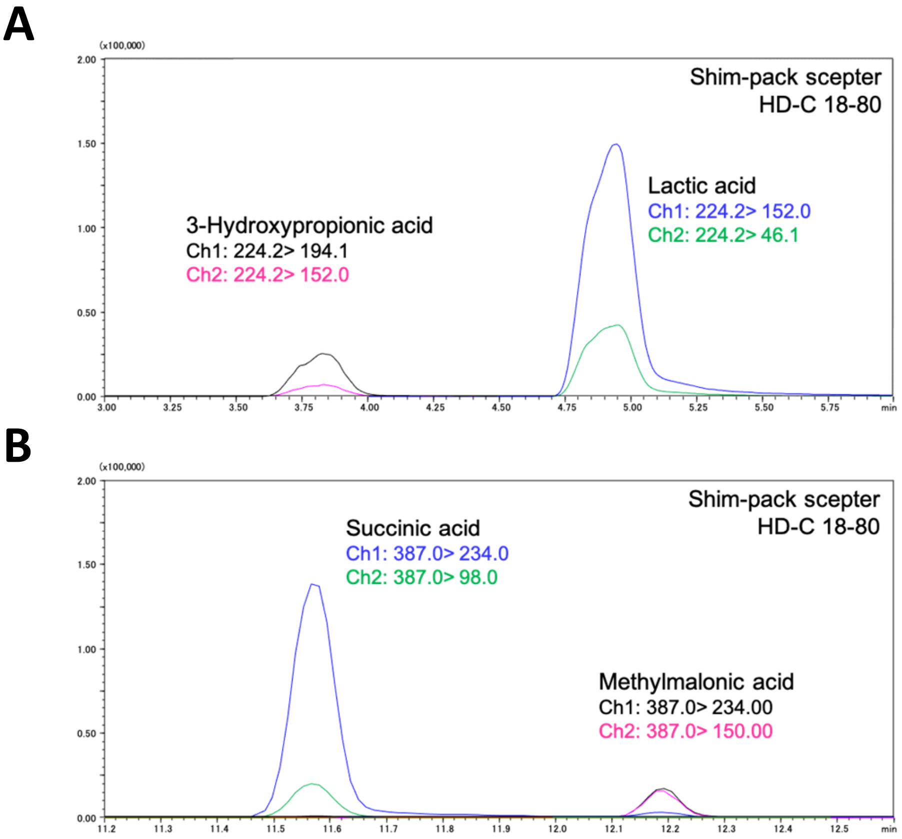

Supplemental Figure 1. Structual isomers separation.
(A) $C_3H_6O_3$: 3-Hydroxypropionic acid and lactic acid. (B) $C_4H_6O_4$: Methylmalonic acid and succinic acid.
